# Distinct ErbB2 receptor populations differentially interact with beta1 integrin in breast cancer cell models

**DOI:** 10.1371/journal.pone.0174230

**Published:** 2017-03-17

**Authors:** Andrés Martín Toscani, Rocío G. Sampayo, Federico Martín Barabas, Federico Fuentes, Marina Simian, Federico Coluccio Leskow

**Affiliations:** 1 Universidad de Buenos Aires, Facultad de Ciencias Exactas y Naturales, Departamento de Química Biológica, Buenos Aires, Argentina; 2 CONICET, Universidad de Buenos Aires, Instituto de Química Biológica de la Facultad de Ciencias Exactas y Naturales (IQUIBICEN), Buenos Aires, Argentina; 3 Universidad de Buenos Aires, Área Investigación, Instituto de Oncología “Angel H. Roffo”, Buenos Aires, Argentina; 4 Centro de Investigaciones en Bionanociencias (CIBION), Consejo Nacional de Investigaciones Científicas y Técnicas (CONICET), Godoy Cruz 2390, C1425FQD Ciudad de Buenos Aires, Argentina; 5 Departamento de Ciencias Básicas, Universidad Nacional de Luján, Luján, Buenos Aires, Argentina; Thomas Jefferson University, UNITED STATES

## Abstract

ErbB2 is a member of the ErbB family of tyrosine kinase receptors that plays a major role in breast cancer progression. Located at the plasma membrane, ErbB2 forms large clusters in spite of the presence of growth factors. Beta1 integrin, membrane receptor of extracellular matrix proteins, regulates adhesion, migration and invasiveness of breast cancer cells. Physical interaction between beta1 integrin and ErbB2 has been suggested although published data are contradictory. The aim of the present work was to study the interaction between ErbB2 and beta1 integrin in different scenarios of expression and activation. We determined that beta1 integrin and ErbB2 colocalization is dependent on the expression level of both receptors exclusively in adherent cells. In suspension cells, lack of focal adhesions leave integrins free to diffuse on the plasma membrane and interact with ErbB2 even at low expression levels of both receptors. In adherent cells, high expression of beta1 integrin leaves unbound receptors outside focal complexes that diffuse within the plasma membrane and interact with ErbB2 membrane domains. Superresolution imaging showed the existence of two distinct populations of ErbB2: a major population located in large clusters and a minor population outside these structures. Upon ErbB2 overexpression, receptors outside large clusters can freely diffuse at the membrane and interact with integrins. These results reveal how expression levels of beta1 integrin and ErbB2 determine their frequency of colocalization and show that extracellular matrix proteins shape membrane clusters distribution, regulating ErbB2 and beta1 integrin activity in breast cancer cells.

## Introduction

ErbB2 is a peculiar member of the ErbB Receptor Tyrosine Kinases (RTKs) family, also known as the Human Epidermal Growth Factor Receptor (HER) or the Epidermal Growth Factor Receptor (EGFR) family, with no identified ligand. ErbB2 is involved in many physiological and pathological processes, such as cancer [1,2]. Its activation is proposed to rely on the interaction with other members of its family [3]. Ligand binding to ErbB induces the formation of ErbB homo- and heterodimers that activate a wide network of secondary messengers [4,5]. In the absence of growth factors, ErbB2 homo-associates in an expression-dependent manner. Size and stoichiometry of membrane clusters of ErbB2 are also determined by the expression levels of other members of the family and the concentration of their ligands [6,7].

ErbB2 overexpression has been described in several types of neoplastic processes, including breast and gastric cancer amongst others [8], and is associated with increased aggressiveness, significantly shortened disease-free and overall survival [9,10]. The anti-ErbB2 antibody trastuzumab (also called Herceptin) [11] is the main therapeutic approach for patients with ErbB2-positive tumors and is thought to lead to downregulation of ErbB2 expression and inactivation of mitogen-activated protein kinase (MAPK) and Akt signaling pathways [12,13]. Understanding ErbB2 interactomics and its regulation is a key factor to improve therapeutical approaches and overcome trastuzumab resistance, a clinical phenomenon that occurs in 66 to 88% of ErbB2-possitive metastatic breast cancers [14].

ErbB2 has been described to interact with several partners in the plasma membrane including proteins involved in cell adhesion such as focal adhesion kinase (FAK), p130Cas, Src and integrins [15–21]. Integrins are heterodimeric receptors for extracellular matrix (ECM) proteins and their specificity is given by the interaction of both alpha and beta subunits [22]. When integrins are activated by ligand binding, they cluster and recruit several proteins forming macromolecular structures called focal adhesion complexes (FAs) [23]. The characteristics of these structures are regulated by both intracellular events, like cytoskeleton rearrangements, and extracellular signals, such as growth factors, chemokines and composition of the ECM [24,25].

Beta1 integrin is a receptor for collagen, laminin and fibronectin (FN) and functions as ECM receptor for cell adhesion and migration, being involved in several physiological and pathological processes [26–28]. Although positive correlation between ErbB2 and beta1 integrin expression has not yet been found [29], beta1 integrin plays a key role in migration and invasiveness of ErbB2-positive breast cancer cells and is involved in trastuzumab resistance [18,30,31]. Cooperative signaling between ErbB proteins and integrins is a common feature of metastatic cancer cells and association between these receptors could provide a framework in which tumor cell migration and invasiveness could be better understood.

Although there is plenty of evidence regarding the crosstalk between ErbB2 and beta1 integrin signaling pathways, the nature of such interaction remains unclear. Formation of heterocomplexes was proven by co-immunoprecipitation assays in cells with high endogenous expression [32] as well as cells transfected with an ErbB2-expression vector [33]. In addition, it has also been demonstrated that ErbB2 and beta1 integrin coexist in membrane clusters of breast and gastric cancer cells in suspension, regardless its sensitivity to trastuzumab [34]. It has been shown by Fluorescence Activated Cell Sorting (FACS)- Förster Resonance Energy Transfer (FRET) that both receptors would be interacting in such membrane clusters of cells with high expression levels of both ErbB2 and beta1 integrin [34,35].

The membrane domains where beta1 integrin and ErbB2 coexist were characterized as lipid rafts [34]. These structures are liquid-ordered membrane microdomains, insoluble in non-ionic detergents at 48°C, rich in cholesterol, sphingomyelin and glycolipids [36,37]. Cell surface receptors are concentrated in lipid rafts which are considered as platforms where signaling machinery converges [38–40]. It has been shown that lipid rafts integrity and dynamics can modulate aggregation, interaction and activation of ErbB receptors [41–43]. Particularly, as ErbB2 is located at lipid rafts, alteration of such structure’s integrity by cholesterol depletion or gangliosides crosslinking with cholera toxin B subunit modifies the homo- and heteroassociation between ErbB2, ErbB3 and EGFR [41,44]. Since beta1 integrin can modulate cholesterol distribution and lipid rafts’s internalization dynamics [45–47], we propose that expression and activation of such ECM receptor would affect the interaction with ErbB2 and thus regulate its function.

The aim of the present work is to study the role of beta1 integrin expression and activation in the context of its interaction with ErbB2. We analyzed the localization of beta1 integrin and ErbB2 in cells with different expression levels plated on FN-coated surfaces as well as in suspension. We determined that beta1 integrin and ErbB2 colocalize only in cells with high expression levels of both receptors when cells are adhered to a FN-coated substrate. In contrast, cells in suspension show coexistence of both receptors in the same membrane domains, suggesting that activation of integrins by FN and its recruitment to FAs would modulate its interaction with ErbB2. Moreover, Stochastic Optical Reconstruction Microscopy (STORM) imaging showed the existence of two populations of ErbB2: a major population located in large clusters and a minor population outside these structures. The minor population is formed by low stoichiometry aggregates and would interact with FA proteins as described in the literature [15–21]. These results reveal the complexity of the ErbB2 interactome and its regulation, showing how the ECM can regulate membrane clusterization and thus the consequent availability of receptors.

## Materials and methods

### Cell culture

MCF7, T47D, SKBR3 and HeLa cell lines were obtained from the American Type Culture Collection (Rockville). A4-HeLa was kindly provided by Dr. Tom Jovin and Dr. Donna Arndt-Jovin (Max Planck Institute for Biophysical Chemistry, Göttingen, Germany). MCF7 and T47D cell lines were routinely maintained in DMEM/F12 cell culture medium (Sigma-Aldrich) supplemented with 10% fetal bovine serum (FBS, Internegocios, Córdoba, Argentina) in a humidified 5% CO2/air atmosphere. SKBR3 cell line was cultured in RPMI medium (Sigma-Aldrich) supplemented with 10% FBS. HeLa and A4-HeLa cell lines were maintained in DMEM high glucose medium (Sigma-Aldrich) supplemented with 10% FBS. Serial passages were carried out by treatment of 80% confluent monolayers with 0.25% trypsin (Invitrogen) and 0.02% EDTA in Ca2+-free and Mg2+-free phosphate-buffered saline (PBS).

### DNA constructs

The coding sequence of murine beta1 integrin was amplified by PCR with Pfu DNA polymerase (Promega) using cDNA from murine neuroblastoma cell line Neuro-2a. The primers used were GCGCTAGCGCTGCGAAAAGATGAATTT (forward) and GCGAATTCATCCGCCTGAGTAGGATTCA (reverse) (Sigma-Aldrich). PCR product was ligated using Nhe I and EcoR I sites into pcDNA 3.1/ZEO(+) (Invitrogen). For stable transfection the coding sequence of beta1 integrin was subcloned into a Geneticin (G418) resistance vector. The insert was subcloned into Nhe I / Not I sites of pECFP-N1 (Clontech), replacing ECFP with the insert after subcloning.

To obtain the ECFP-fused version, it was used a reverse primer designed in order to codify an 18 aminoacid linker (GGGGARRRGQAGDPPVAT) between beta1 integrin and the fluorescent protein [48]: CGGGATCCCCAGCTTGTCCTCGTCGTCGAGCTCCTCCTCCTCCTTTTCCCTCATACTTCGGATT (Sigma-Aldrich). PCR product was ligated using Nhe I and BamH I sites into pcDNA 3.1/ZEO(+) (Invitrogen). ECFP was extracted from pECFP-N1 (Clontech) and ligated into BamH I / Not I sites. In order to generate the beta1 integrin-ECFP shRNA 3’UTR construct, IRES and shRNA sequences was subcloned from shItgb1-T [49] and inserted into Pme I / Not I sites.

ErbB2-TagRFP was obtained by subcloning ErbB2 from ErbB2-mYFP (Dr. Tom Jovin and Dr. Donna Arndt-Jovin, Max Planck Institute for Biophysical Chemistry, Göttingen, Germany) into Nhe I / HinD III sites of pTagRFP-N (Evrogen).

Alpha6 Integrin expression vector was kindly provided by Dr. Arnoud Sonnenberg (Netherlands Cancer Institute, Amsterdam, The Netherlands). All constructs were verified by sequencing.

### Transfection

MCF7, HeLa and HC11 cells were transfected using Lipofectamine 2000 (Invitrogen) according to the manufacturer’s protocol. Selection of stable transfectants was performed three days after transfection. Cells were cultured in complete media with 700 ug/ml of G418 (Sigma-Aldrich) for 16 days. 300 ug/ml of G418 was used for maintenance of selected cells. Transfectants were grown in G418-free medium for at least 7 days before experiments.

### Antibodies

OP15 mouse monoclonal antibody against an intracellular epitope of ErbB2 (Calbiochem-Merck Biosciences) was used 1:1000 for Western blot and 1:500 for immunofluorescence. trastuzumab-Alexa Fluor 488 (kindly provided by Dr. Tom Jovin and Donna Arndt-Jovin, Max Planck Institute for Biophysical Chemistry, Göttingen, Germany) was used 5 ng/uL for immunofluorescence. MAB1981 (LM534, EMD Millipore) mouse monoclonal non-blocking antibody against an extracellular epitope of beta1 integrin was used 1:500 for immunofluorescence. D2E2 (mAb9699, Cell Signaling Technology) rabbit monoclonal antibody for beta1 integrin was used 1:1000 for Western blot. C-4 (Santa Cruz Biotechnology) polyclonal antibody against beta-actin was used 1:5000 for Western blot. G-9 (Santa Cruz Biotechnology) polyclonal antibody against GADPH was used 1:2000 for Western blot.

Goat anti-mouse HorseRadish Peroxidase (HRP) conjugated and goat anti-rabbit HRP conjugated (Genetech) were used 1:5000 for Western blotting. Goat anti-mouse Alexa Fluor 488 and Alexa Fluor 647-conjugated antibodies (Life Technologies) were used 1:600 for immunofluorescence.

### Western blot

Whole cell protein extracts were prepared by scrapping the culture dishes on ice with RIPA buffer (50mM Tris, pH 8.0 containing 150 mM NaCl, 0.1% SDS, 0.5% deoxycholate and 1% NP40) containing protease and phosphatase inhibitors (40 uM phenylmethylsulfonyl fluoride, 5 ug/ml leupeptin, 50 ug/ml aprotinin, 40 mM sodium fluoride and 100 mM beta-glycerophosphate). Protein concentration was measured by Bradford. 50 ug of each sample were then run in SDS-PAGE mini gels and transferred to PVDF membranes (Amersham Biosciences). Membranes were blocked for 1 hour at R.T. in 5% bovine serum albumin (BSA) in Tris-Buffered Saline plus 0.1% Tween-20 (TBST). Primary antibodies were prepared in blocking buffer and incubated at 4°C ON. After washing 5 times with TBST, membranes were incubated with secondary antibodies for 1 hour at RT and washed 5 times with TBST. Signal was detected with an enhanced chemiluminescence kit (ECL, Amersham Biosciences).

### Immunofluorescence

Fifty thousand cells were seeded on 12 mm glass coverslips (Marienfeld) in 24-well plates. Coverslips were first coated with Poli-D-Lysine (Sigma-Aldrich) 40 ug/ml in 0.1 M borate buffer for 1 hour at 37°C, washed with PBS and subsequently incubated 1 hour at 37°C with laminin I (Sigma-Aldrich) 20 ug/ml or FN (Invitrogen) 50 ug/mL in PBS.

After 16 hours, cells were starved 4 hours with FBS-free medium, washed with PBS and fixed 10 minutes at RT with 4% paraformaldehyde (PFA) 4% sucrose in PBS. PFA was quenched by incubation with 10mM Tris-PBS 5 minutes at RT. Fixed cells were incubated in blocking buffer (0.1% Triton-X100. 5% BSA in PBS) 1 hour at 4°C. Primary antibodies diluted in blocking buffer were incubated for 1 hour at 4°C and washed 5 times with PBS. Secondary antibodies were incubated in blocking buffer for 1 hour at 4°C and washed 5 times with PBS. Samples were mounted in Mowiol-based mounting media.

For immunofluorescence of cells in suspension, cells were treated with 100mM EDTA PBS to induce detachment and transferred to a 1.5 mL tube. Cells were washed by centrifugation 5 minutes at 9000 rpm and preceded as previously described. After washing the secondary antibody, cells were resuspended in PBS and placed in a PDL-coated 8-well LabTec chamber (Nunc/Thermo Fisher Scientific) for imaging.

### Confocal microscopy

Confocal laser scanning microscopy (CLSM) images were acquired in an Olympus FV1000 microscope (Olympus) using an Olympus 60x / 1.42NA UPLAN SAPO oil immersion objective. Excitation and filters were as follows: Alexa Fluor 488: excitation 488 nm Argon laser line—emission 505–525 nm BP filter. TagRFP: excitation 543 nm Helium/Neon laser—emission 560–620 nm BP filter. Alexa Fluor 647: excitation 633 nm diode laser—emission 650–750 nm BP filter. ECFP: excitation 440 nm pulsed laser excitation- emission 470–490 nm band pass (BP) filter. EYFP: excitation 515 nm Argon laser line—emission 525–535 nm BP filter. Images were acquired in a sequential mode. Bleedthrough was checked by imaging of samples labeled with a single fluorophore and acquiring dual channel images with the same setup used for the co-labeled system.

### Colozalization analysis

Quantification of Pearson’s correlation, Manders’s overlap, M1 and M2 coefficients was performed using Villalta’s algorithm [50]. This algorithm runs on Matlab (MathWorks) using the image processing toolbox DIPimage (Delft University of Technology). We used the Costes thresholding method to determine the threshold values [51]. The maps for these coefficients were computed by estimating the contribution of each single pixel to the coefficient.

### STORM imaging

The STORM microscope was built around an Olympus IX-71 inverted microscope operating in wide-field epifluorescence mode. A 640 nm 75 mW laser was used for fluorescence excitation and a 405 nm 53 mW laser for fluorescence re-activation, both from RGB Lasersystems. The lasers were combined with a dichroic mirror (CM01-427, Semrock), magnified and then focused to the back focal plane of the oil immersion objective Olympus PlanApo 60x NA 1.42. A dichroic mirror (Di02-R635, Semrock) and a long-pass filter (ET655lp, Chroma) were used for decoupling of the fluorescence emission of the sample from the laser excitation and further filtering was performed with 405 nm and 633 nm notch filters from Semrock. The diffraction-limited images were recorded with an Andor 885 EMCCD camera attached to the left side port of the microscope’s stand. The camera and the lasers control were controlled with the manufacturer’s software. Data analysis was performed with DAOSTORM algorithm [52] using 3D-DAOSTORM software from ZhuangLab online repository.

Cells were cultured in 18 mm coverslips, fixed and labeled using OP15 antibody (Millipore, Germany) as previously described. Alexa Fluor 647-conjugated secondary antibody (Life Technologies) was used. Samples were placed in a holder and imaging was performed in a 50 mM Tris pH = 8, 10 mM NaCl buffer. Imaging buffer was supplemented with 10% w/v glucose, 100mM beta-mercaptoethanol, 1 ug/mL glucose oxidase (Sigma-Aldrich) and 0.5 ug/mL catalase (Sigma-Aldrich) as an oxygen scavenging system. The oxygen scavenging system is critical for reliable photoswitching of the fluorophores [53].

Prior to STORM imaging, conventional fluorescence images of the region of interest were acquired by setting the excitation laser intensity to 1–5 W/cm2. STORM data acquisition was then started by changing the excitation laser intensity to 1–5 kW/cm2, thus inducing on-off switching of the fluorescent marker in the tens of ms time range, as required by the STORM technique. During the whole acquisition, the activation laser power was increased in steps whenever the density of single-molecule events decreased below ~1 molecule per um2. Typically, it took 10000–20000 frames at 50 ms of exposition time for each STORM acquisition.

### Bioinformatical analysis

mRNA expression data published by TCGA [54] was obtained from cBioPortal Cancer Genomics Portal [55].

### Statistical analysis

All statistical analyses were carried out using Prism 5 (GraphPad Software). Tests and sample number used are indicated in the figures legends.

## Results

### Expression and localization of beta1 integrin and ErbB2

**Fig 1:**
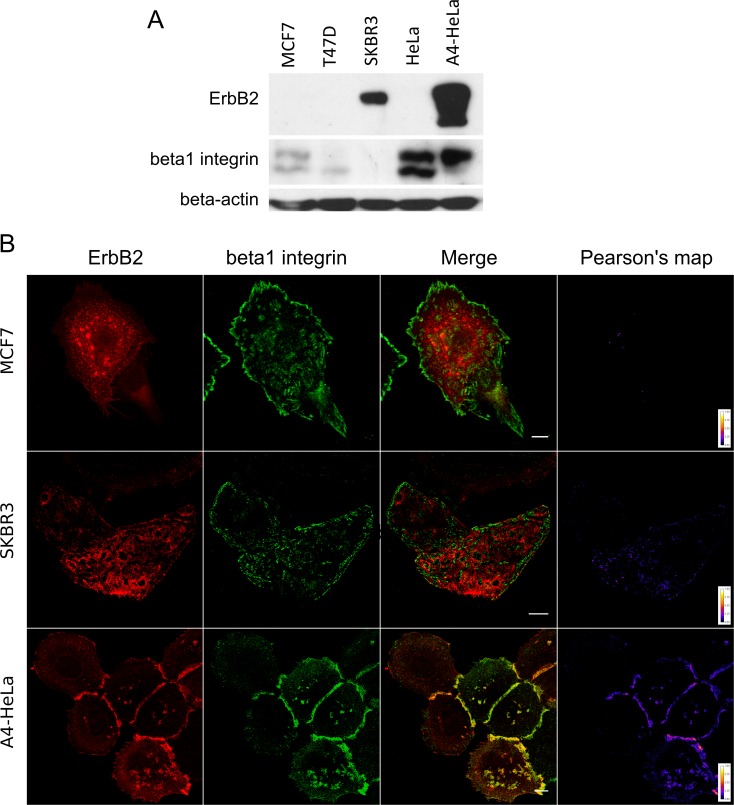
Coexistence of ErbB2 and beta1 integrin depends on expression levels. (A) Western blot for ErbB2 and beta1 integrin of MCF7, T47D, SKBR3, HeLa y A4-HeLa cells (HeLa cells stably expressing the ErbB2-mYFP fusion protein). As MCF7 cells express very low levels of ErbB2 (not detected by immunolabeling) it was necessary to transfect them with the ErbB2-TagRFP construct in order to perform fluorescence imaging. (B) CLSM images of MCF7, SKBR3 and A4-HeLa plated on FN-coated coverslips. It was not possible to observe colocalization between ErbB2 and beta1 integrin in both MCF7 and SKBR3 cells. In contrast, A4-HeLa cells (which express high levels of both these receptors) show higher colocalization levels in the plasma membrane as shown in the Pearson’s correlation coefficient’s map. Scale bar: 10um.

Using cancer cell lines that differ in their expression levels of beta1 integrin and ErbB2, we used confocal imaging to evaluate the localization of these proteins. Breast cancer cell line MCF7 showed non-detectable levels of ErbB2 and moderate levels of beta1 integrin, the main FN receptor expressed in this cell line [56] (Fig 1A). Due to their low ErbB2 expression levels, undetectable by immunofluorescence, we generated a pool of cells stably expressing the fusion protein ErbB2-TagRFP. We used SKBR3, a Herceptin-sensitive breast cancer cell line, as a high ErbB2 / low beta1 integrin cell model (Fig 1A). In addition, we used a HeLa-derived cell line that presents high beta1 integrin and ErbB2 expression (A4-HeLa, Fig 1A). This cell line stably expresses the ErbB2-mYFP fusion protein allowing direct imaging of ErbB2 [6].

Cells expressing low levels of beta1 integrin (MCF7 and SKBR3) show no colocalization between ErbB2 and beta1 integrin on a FN-coated surface (Figs 1B and 3B). ErbB2 forms large clusters in the plasma membrane of variable size. Such structures are located outside FAs formed by association of integrins upon activation by FN. In contrast, A4-HeLa cells, that present high levels of beta1 integrin, show high colocalization rates between these two receptors (Figs 1B and 3B). Although ErbB2 forms large clusters in the same fashion as in MCF7 and SKBR3, beta1 integrin is diffusely localized at the plasma membrane, forming small FAs and coexisting with ErbB2 in these large macromolecular complexes (Fig 1B).

### Overexpression of beta1 integrin and ErbB2 induces coexistence of these receptors in membrane clusters

As A4-HeLa cells show a strong colocalization between beta1 integrin and ErbB2, we studied if such coexistence is a particular feature of this cell line or it is a consequence of the high expression levels of both receptors. To this end, we transiently overexpressed ErbB2-mYFP and beta1 integrin-ECFP in HeLa cells.

**Fig 2:**
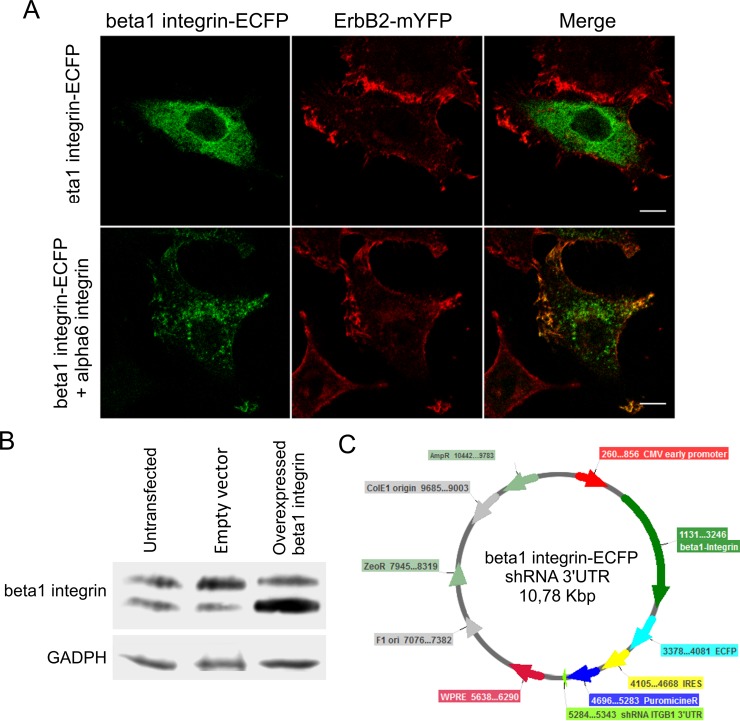
Overexpression of beta1-integrin in cultured cells. (A) CLSM image of an A4-HeLa cell transfected with the beta1 integrin-ECFP construct. The fluorescent protein signal could be detected homogeneously distributed in the cytoplasm. When A4-HeLa were transfected with beta1 integrin-ECFP and alpha6 integrin expression vectors and plated on laminin-coated coverslips, membrane structures could only be identified in cells with low expression levels of ECFP signal. (B) Western blot of MCF7 cells stably-transfected with a beta1 integrin expression vector. Cells expressing exogenous beta1 integrin show higher levels of the low molecular weight band of beta1 integrin. (C) Plasmid map of beta1 integrin-ECFP shRNA 3’UTR. This construct has the coding sequence of beta1 integrin-ECFP followed by an IRES sequence and a shRNA targeted to the 3’UTR of murine beta1 integrin mRNA. This plasmid allows us to simultaneously diminish the endogenous levels of beta1 integrin while the fusion protein is overexpressed.

Cells expressing beta1 integrin-ECFP fusion protein showed high cytoplasmatic signal for ECFP rather than membrane localization (Fig 2A). This might be due to the complex regulation of beta1 integrin, including posttranslational modifications and the need of partnering with an alpha subunit to be functional [22,57,58]. Therefore, we co-transfected beta1 integrin-ECFP with an alpha6 integrin construct in HeLa cells to promote membrane localization of alpha6/beta1 integrin dimers. Since alpha6/beta1 integrin dimers are specific receptors for laminin [22,59], we plated these cells on laminin-coated coverslips to induce activation of these ECM receptors. However, only cells with low expression levels of beta1 integrin showed membrane localization of ECFP signal and formation of clusters (Fig 2A). A possible explanation to this phenomenon is that overexpression of beta1 integrin would saturate the post translational modification machinery and stock the extra -immature- protein in the endoplasmic reticulum or Golgi apparatus. In accordance with these results, MCF7 cells stably expressing exogenous beta1 integrin showed hardly any increase in the levels of the mature integrin but the expression of a lower molecular weight form was increased (Fig 2B). This lighter band corresponds to the non-glycosylated form of beta1 integrin, stored in the Golgi apparatus [60,61]. It has been proved that integrin glycosylation is crucial for its dimerization, ligand binding and localization [57,58]. Therefore, alterations in this post translational modification impairs integrin clusterization and FAs formation. This is consistent with previous reports showing that beta1 integrin-EGFP could only be expressed and properly targeted to FAs in beta1 integrin knock-out cells [48].

**Fig 3:**
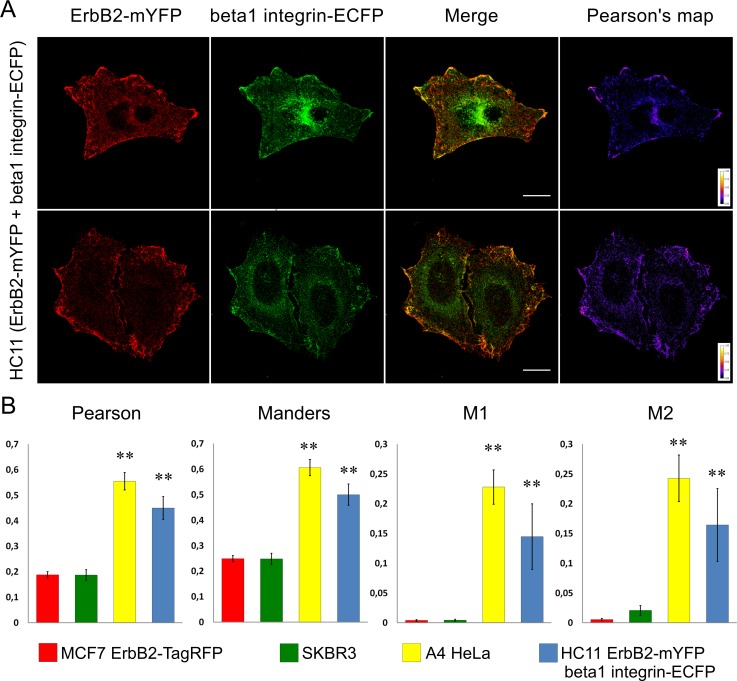
ErbB2 and beta1 integrin coexist in membrane clusters of cells with overexpressed receptors. (A) HC11 cells transfected with beta1 integrin-ECFP-shRNA and ErbB2-mYFP plasmids plated on FN-coated coverslips. Similar to A4-Hela, it is possible to observe membrane structures where both proteins colocalize in a very reproducible fashion. (B) Quantification of Pearson’s correlation, Manders’ overlap, M1 and M2 coefficients for MCF7 (red bars), SKBR3 (green bars), A4-HeLa (yellow bars) and HC11 cotransfected with beta1 integrin-ECFP-shRNA and ErbB2-mYFP plasmids (blue bars). Scale bar: 10um. ** p<0.01 vs. MCF7 and SKBR3 (ANOVA followed by Tukey's test), n = 23 for MCF7, SKBR3 and A4-HeLa, n = 15 for HC11. Data set available in S1 Fig.

In order to successfully express beta1 integrin-ECFP at the plasma membrane, we developed a novel strategy to express exogenous beta1 integrin while knocking down its endogenous expression. For this purpose we cloned an IRES driven shRNA sequence targeted to the 3’UTR of the murine transcript downstream beta1 integrin-ECFP coding sequence (Fig 3C). This shRNA was developed and characterized by J. Naipauer and collaborators [49]. This resulting construct, named “beta1 integrin-ECFP shRNA 3’UTR”, was transfected into murine HC11 cells, derived from normal mammary gland. We obtained a clustered localization of the ECFP signal at the plasma membrane (Fig 3A). The size and location of these clusters are similar to those observed in previously reported beta1 integrin knock-down cells transfected with beta1 integrin-EGFP [48]. This construct provided us with a great advantage as it allowed the expression of a fully functional beta1 integrin-ECFP in murine cells regardless of the endogenous beta1 integrin levels.

In order to study the colocalization of beta1 integrin-EGFP and ErbB2, HC11 murine normal mammary epithelial cells were transfected with beta1 integrin-ECFP shRNA 3’UTR and ErbB2-mYFP expression vectors. Consistent with the results observed for A4-HeLa, overexpressed receptors colocalize in large clusters at the cell membrane (Fig 3A). These results suggest that beta1 integrin and ErbB2 coexistence in membrane clusters is highly dependent on beta1 integrin levels. High expression of beta1 integrin might leave some of these receptors unbound to extracellular proteins and available to interact with ErbB2 in large clusters, explaining why they interact even when cells are seeded on FN (Fig 3B). In cells with low or moderate expression of beta1 integrin plated on FN, this protein is exclusively located in FAs and does not colocalize with ErbB2 (Fig 3B).

### Expression of beta1 integrin correlates with ErbB2 only in Her2-possitive breast invasive ductal carcinoma

**Fig 4:**
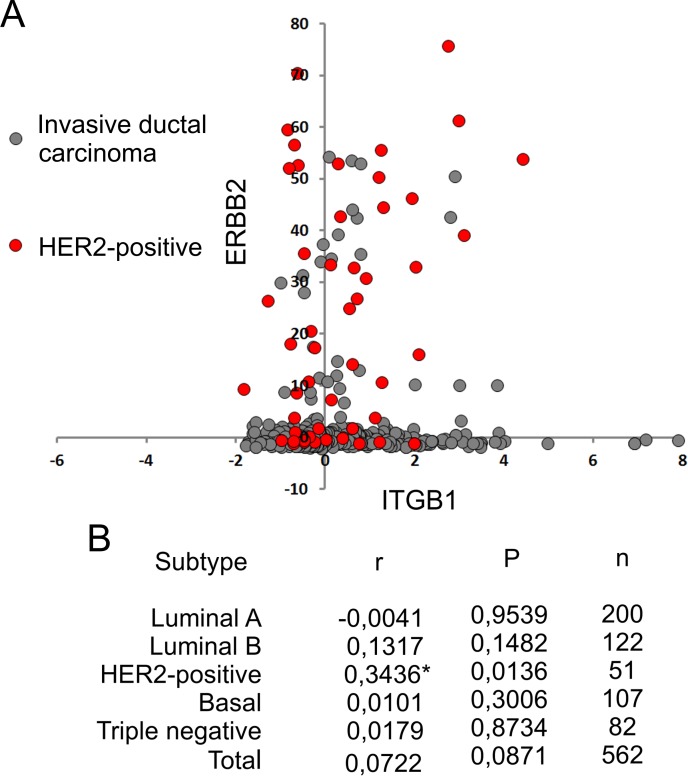
Coexpression of ErbB2 and beta1 integrin in breast cancer. (A) Dot-plot of mRNA expression Z-Scores for breast cancer samples published by TCGA in 2015 [60] with PAM50 characterization (black dots) and HER2-positive tumors (red dots). (B) Pearson´s correlation coefficient (r), two-tailed P value (P) and number of samples (n) for different invasive ductal carcinomas subtypes. *r values regarded as significant correlation (alpha = 0.05).

ErbB2 is particularly relevant in invasive breast carcinoma as its enrichment results in poor prognosis and shortened survival [62]. To further investigate the clinical relevance of ErbB2 and beta1 integrin expression levels and their interaction, we studied the coexpression of such receptors in breast cancer samples from patients. We analyzed the correlation of ErbB2 and beta1 integrin expression in RNAseq data published by TCGA in 2015 [54] (Fig 4). We observed a positive correlation between ErbB2 and beta1 integrin mRNA expression only in HER2-positive breast invasive ductal carcinomas (characterized by high expression levels of ErbB2 [63]), supporting the data obtained in cell culture models. As high expression of beta1 integrin induces coexistence of these receptors in membrane clusters, beta1 integrin expression would represent an adaptive advantage for ErbB2-positive metastatic cells by facilitating receptors interaction and cross talk between signaling pathways and thus enhancing their metastatic capacity.

### Lack of ligand-binding by beta1 integrin promotes clusterization with ErbB2

Ligand binding to a membrane receptor behaves as a chemical equilibrium ruled by an affinity constant. If the concentration of such receptors in the membrane increases dramatically, but not the amount of ligand, more of them would remain unbound and inactive. In cells with low or intermediate amounts of beta-1 integrin, like MCF7 and SKBR3, this receptor localizes in FAs. In contrast, cells with high expression of this protein show a more diffuse distribution of beta1 integrin. Overexpression of integrins may increase the amount of ECM-unbound integrins as the competition for the ligand binding domain (like the RGD sequence of FN) is higher. This would leave more receptors delocalized and available to interact with ErbB2. To test this hypothesis, we asked whether the same effect observed by increasing beta1 integrin expression could be achieved by promoting integrin detachment from their ECM ligands. We cultured MCF7 cells stably expressing the fusion protein ErbB2-TagRFP and detached them by treating with 10mM EDTA in PBS. This strategy allows cell detachment without proteolytic cleavage of membrane proteins. Cells in suspension were immunolabeled for beta1 integrin and imaged by CLSM.

**Fig 5:**
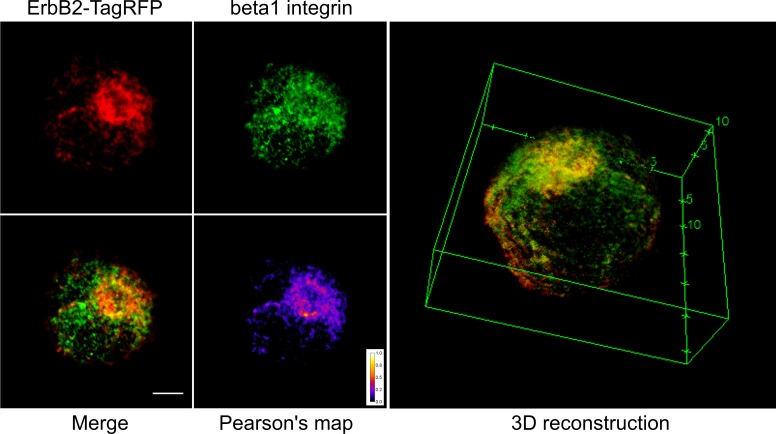
Immunolabeling of MCF7 cells in suspension expressing the fusion protein ErbB2-TagRFP. MCF7 cells expressing the fusion protein ErbB2-TagRFP in suspension were immunolabeled for beta1 integrin. 3D-reconstruction allows us to determine that both proteins coexist in conspicuous domains in the surface of the cell membrane. This is a representative image of 20 acquired cells. All cells showed clusters with positive colocalization (r > 0.5). Scale bar: 5um.

Although cells plated on FN-coated coverslips showed no colocalization between ErbB2 and beta1 integrin (Fig 1B), when cells are in suspension both proteins colocaize in conspicuous structures in the membrane (Fig 5). Three-dimensional reconstruction shows that such clusters are present in the entire cell surface (Fig 5), consistent with the results published by Mocanu and collaborators using cells in suspension for FACS analysis of ErbB2 and beta1 integrin interaction [34]. Consistent with the results presented before (Fig 3B), these data show that not only beta1 integrin expression but also activation by ECM proteins regulate beta1 integrin localization and thus its interaction with ErbB2. In contrast, activation of ErbB2 by HRG or EGF showed no significant differences in its interaction with beta1 integrin (data not shown).

### Superresolution microscopy shows two different populations of ErbB2 in the cell membrane

ErbB2 spontaneously forms homo-clusters in the membrane. These dynamic structures are regulated by ligand binding and expression levels. As it was previously described, there is an equilibrium between monomers and higher order aggregates of ErbB2 in the membrane [6,7]. Single particle tracking experiments showed that ErbB monomers have two different behaviors: monomeric receptors can be confined in membrane domains or moving freely in the plasma membrane [64], suggesting that there should also be an equilibrium between monomers inside and outside these membrane domains.

**Fig 6:**
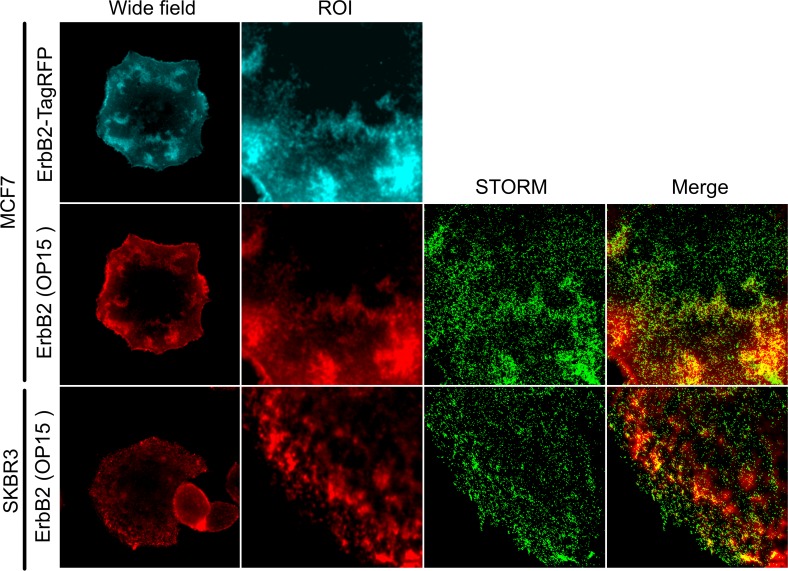
STORM images of MCF7 expressing the ErbB2-TagRFP fusion protein and SKBR3 cells. Cells were labeled with a specific antibody for ErbB2 (OP15, Millipore) and a secondary antibody coupled with the Alexa Fluor 647 fluorophore. It was possible to determine the presence of two different receptor populations: one inside the clusters and another outside these structures, seen as small spots, representing monomers or low stoichiometry aggregates of ErbB2.

In order to further study the ErbB2 clusters in our model system, STORM imaging was performed. This technique allows us to study the morphology of these structures in greater detail as it has a one order of magnitude higher resolution than CLSM. To generate STORM images, indirect immunofluorescence was performed using the anti-ErbB2 antibody OP15 (Millipore) together with a secondary antibody coupled to the fluorophore Alexa Fluor 647. Although this labeling approach didn’t allow us differentiate between monomers and low stoichiometry aggregates, it was possible to observe receptors outside large clusters in both MCF7 cells stably expressing ErbB2-TagRFP and SKBR3 cells plated on FN-coated coverslips (Fig 6).

These results allowed us to infer that there is a minority non-clustered ErbB2 population outside large clusters that are undetectable with conventional fluorescence microscopy techniques. The low amount and surface distribution of this non-clustered population represents very low signal in CLSM so they do not contribute to the correlation coefficients previously calculated (Fig 3B). Although they represent a low proportion of receptors in the membrane, these non-clustered ErbB2 molecules could be interacting with proteins in the FAs such as p130CAS or FAK, as described in the literature [15–21]. Our data reveal for the first time the existence of a minor population of ErbB2 within the cell membrane. We hypothesize that this population could interact with FAs even though large clusters of erbB2 do not colocalize with this structures. In this model system, this minor population of erbB2 would account for the interaction with FA proteins that has been described by others using biochemical approaches.

## Conclusions

Cell membranes are complex and dynamic structures. They are compartmentalized in domains with different physicochemical and biological features. This organization defines regions where functionally related proteins are assembled in order to achieve more efficient biological processes such as signal transduction, cell adhesion and electron transport in a respirasome, among other.

Plenty of evidence suggests that there is a close interaction between molecules involved in cell adhesion and RTKs. Particularly, ErbB2 has been proposed to interact with integrins, FAK and p130CAS, amongst others [15–21]. Beta1 integrin has become relevant due to its ubiquitous expression and involvement in several cell processes such as migration, polarization of epithelia and differentiation of many tissues [26]. In addition, this protein is involved in many pathological processes, particularly in cancer [65]. In this context, interplay between beta1 integrin and ErbB2 would play a major role in tumor growth and metastasis [30,66].

**Fig 7:**
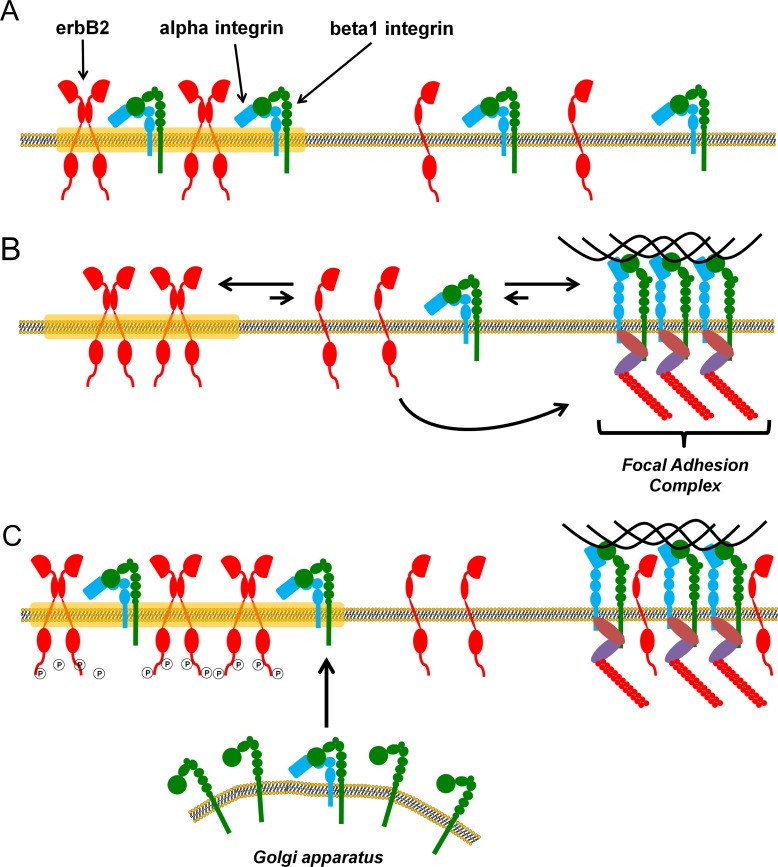
Model proposed for ErbB2 and beta1 integrin interaction dynamics in the cell membrane. (A) When cells are not attached to the substrate, ErbB2 and beta1 integrin coexist in membrane clusters described as lipid rafts by Mocanu and collaborators. (B) When integrins are activated by a ligand, such as FN, they leave the ErbB2 clusters and recruit other protein forming the FAs. Although there is no colocalization between ErbB2 clusters and FAs, ErbB2 molecules outside these structures could interact with FAs proteins such as FAK or p130CAS as described in the literature. (C) When ErbB2 and beta1 integrin are overexpressed the system is saturated and the equilibrium between free, FAs and ErbB clusters-linked beta1 integrin is disrupted. In this condition, overexpression of ErbB2 has been found to induce autophosphorylation and ligand-independent activation of signaling pathways such as Akt or MAPK. In this context, self-activation of ErbB2 could lead to FA protein phosphorylation as they could be physically close to each other due to beta1 integrin location in ErbB2 clusters.

There is evidence suggesting a functional interaction between ErbB2 and beta1 integrin at the plasma membrane although the nature of such interaction remains unclear [34,35]. In this work, we present data showing that this coexistence occurs in very restricted situations. We propose a model that brings consensus to the contradictory data shown in the literature. When cells are in suspension, inactive beta1 integrin and ErbB2 coexist in conspicuous structures across the entire cell surface (Fig 7A). Upon integrin activation by ECM proteins, beta1 integrin rearranges to form FAs losing its location in ErbB2 clusters (Fig 7B). This is consistent with reports showing that beta1 integrin-mediated cell adhesion regulates membrane domains and lipid distribution at the membrane [47,67]. In the context of metastatic tumor cells, FAK and beta1 integrin are key players in the outside-in signaling necessary for colonization of new environments during metastasis [30,66,68]. We propose that this could also enhance ErbB2 signaling as both receptors are in the same clusters when cells are unattached. Furthermore, in adherent cancer cells, elevated beta1 integrin and ECM proteins would induce rearrangement of ErbB2 membrane domains and receptor redistribution after the cell adheres to the substrate [69].

As shown by STORM imaging (Fig 6), ErbB2 monomers or low-stoichiometry oligomers are outside ErbB2 clusters. These receptors would be able to diffuse, interact and activate signaling proteins in FAs, as it was shown in the literature [15–19]. On the contrary, receptors located in large clusters cannot interact with such structures (Fig 7B). Although it is not possible to rule out the existence of receptors in the mature FAs, they cannot be detected by the techniques here used here. On the other hand, when both ErbB2 and beta1 integrin are overexpressed, equilibrium between free monomers and clustered receptors is altered and it is possible to observe ErbB2 and beta1 integrin coexistence in large clusters across the membrane (Fig 7C). Under these conditions, functional interaction between these two receptors could be promoted. In addition, overexpression of ErbB2 promotes ligand-independent phosphorylation and receptors self-activation. As beta1 integrin is placed in these clusters, ErbB2 would be closely linked to the FAs signaling machinery, inducing its phosphorylation and thus enhancing FAs signaling as well.

Huck and collaborators proved that lack of beta1 integrin expression in ErbB2-positive breast cancer cells impairs proliferation, blood vessel formation and decreases lung metastasis by inhibiting Src, FAK, p130CAS and Paxillin phosphorylation [30]. These findings highlight the importance of beta1 integrin expression during ErbB2-driven metastasis. Our results provide a better understanding of the necessary conditions for ErbB2 and beta1 integrin to coexist in membrane clusters. Such interaction would give metastatic ErbB2-positive cancer cells a framework for them to survive without ECM attachment. Consistently, bioinformatic analysis showed that HER2-positive breast invasive ductal carcinomas are the only subtype having a positive correlation between ErbB2 and beta1 integrin mRNA expression (Fig 4). All together, these results support the hypothesis that high expression of beta1 integrin would represent an adaptive advantage to ErbB2-positive metastatic cells.

As we showed, interaction between ErbB2 and beta1 integrin is a complex, dynamic and multifactorial process that involves many variables: protein expression, phosphorylation levels, presence and concentration of partner molecules or ligands, among others. Despite its complex regulation, the interaction between ErbB2 and beta1 integrin signaling pathways plays a key role in many physiological and pathological processes. In particular, it would have a major role in HER2-positive subtypes, which represents 20 to 30% of breast cancers and still remains the second most aggressive and with the lowest overall survival. Further understanding of ErbB2 interactome, activation dynamics and crosstalk with other signaling pathways would help to develop new therapeutic approaches to overcome this disease.

## Supporting information

S1 Fig Quantification of ErbB2 and beta1 integrin colocalization. Quantification of Pearson’s correlation, Manders’ overlap, M1 and M2 coefficients of individual images of MCF7, SKBR3, A4-HeLa and HC11 cells cotransfected with beta1 integrin-ECFP-shRNA and ErbB2-mYFP plasmids.
(PDF)
