# Supplementary material for: Distinct ErbB2 receptor populations differentially interact with beta1 integrin in breast cancer cell models
Source: PLoS One. 2017 Mar 17;12(3):e0174230. doi: 10.1371/journal.pone.0174230 (PMC5357064; doi:10.1371/journal.pone.0174230)
Supplement: S1 Fig — Quantification of Pearson’s correlation, Manders’ overlap, M1 and M2 coefficients of individual images of MCF7, SKBR3, A4-HeLa and HC11 cells cotransfected with beta1 integrin-ECFP-shRNA and ErbB2-mYFP plasmids. (PDF) [file pone.0174230.s001.pdf]

	MCF7 ErbB2-TagRFP				SKBR3				A4-HeLa				HC11 ErbB2-mYFP beta1 integrin-ECFP			
	Pearson	Manders	M1	M2	Pearson	Manders	M1	M2	Pearson	Manders	M1	M2	Pearson	Manders	M1	M2
	0.14	0.22	0.00	0.00	0.21	0.27	0.00	0.01	0.42	0.49	0.09	0.09	0.52	0.57	0.23	0.21
	0.20	0.26	0.00	0.01	0.12	0.16	0.00	0.00	0.69	0.74	0.27	0.32	0.48	0.51	0.18	0.19
	0.12	0.16	0.00	0.00	0.21	0.28	0.00	0.00	0.73	0.75	0.21	0.32	0.33	0.42	0.00	0.00
	0.07	0.13	0.00	0.00	0.11	0.18	0.00	0.00	0.53	0.58	0.25	0.35	0.32	0.38	0.00	0.00
	0.25	0.29	0.02	0.04	0.07	0.13	0.00	0.00	0.48	0.54	0.12	0.19	0.54	0.59	0.25	0.41
	0.15	0.20	0.00	0.00	0.09	0.22	0.00	0.00	0.69	0.71	0.30	0.30	0.27	0.32	0.00	0.00
	0.24	0.28	0.00	0.00	0.22	0.29	0.00	0.01	0.38	0.43	0.12	0.16	0.62	0.65	0.43	0.43
	0.24	0.27	0.00	0.00	0.16	0.24	0.00	0.01	0.45	0.51	0.07	0.04	0.51	0.56	0.06	0.12
	0.12	0.19	0.00	0.00	0.18	0.29	0.00	0.00	0.55	0.58	0.19	0.21	0.69	0.75	0.36	0.29
	0.20	0.26	0.00	0.00	0.14	0.21	0.00	0.01	0.47	0.54	0.19	0.10	0.56	0.80	0.21	0.23
	0.14	0.22	0.00	0.00	0.16	0.25	0.00	0.00	0.67	0.72	0.21	0.19	0.60	0.72	0.21	0.15
	0.19	0.24	0.00	0.00	0.22	0.32	0.00	0.00	0.70	0.84	0.47	0.38	0.35	0.46	0.00	0.00
	0.13	0.19	0.00	0.00	0.22	0.33	0.01	0.04	0.61	0.60	0.25	0.28	0.34	0.35	0.00	0.00
	0.29	0.34	0.00	0.00	0.42	0.48	0.03	0.15	0.76	0.81	0.50	0.45	0.62	0.65	0.22	0.33
	0.20	0.31	0.00	0.00	0.30	0.38	0.00	0.02	0.72	0.85	0.37	0.41	0.48	0.51	0.04	0.06
	0.21	0.26	0.00	0.00	0.28	0.32	0.01	0.12	0.64	0.67	0.27	0.19				
	0.05	0.10	0.00	0.00	0.14	0.16	0.00	0.02	0.71	0.75	0.40	0.38				
	0.16	0.25	0.01	0.00	0.07	0.09	0.00	0.00	0.51	0.60	0.29	0.32				
	0.23	0.30	0.03	0.03	0.09	0.13	0.00	0.00	0.59	0.54	0.20	0.26				
	0.25	0.31	0.00	0.01	0.08	0.11	0.00	0.00	0.51	0.54	0.21	0.32				
	0.22	0.27	0.01	0.01	0.41	0.43	0.02	0.07	0.35	0.45	0.09	0.06				
	0.23	0.31	0.00	0.00	0.11	0.15	0.00	0.00	0.43	0.49	0.05	0.03				
	0.22	0.27	0.02	0.02	0.10	0.13	0.00	0.00	0.63	0.62	0.27	0.30				
**Average**	0.18	0.25	0.00	0.01	0.18	0.24	0.00	0.02	0.57	0.62	0.23	0.25	0.48	0.55	0.15	0.16
**SD**	0.06	0.06	0.01	0.01	0.10	0.10	0.01	0.04	0.12	0.13	0.12	0.12	0.13	0.15	0.14	0.15
**n**	23	23	23	23	23	23	23	23	23	23	23	23	15	15	15	15
**SEM**	0.01	0.01	0.00	0.00	0.02	0.02	0.00	0.01	0.03	0.03	0.03	0.03	0.03	0.04	0.04	0.04

Fig S1. Quantification of ErbB2 and beta1 integrin colocalization.

Quantification of Pearson's correlation, Manders' overlap, M1 and M2 coefficients of individual images of MCF7, SKBR3, A4-HeLa and HC11 cells cotransfected with beta1 integrin-ECFP-shRNA and ErbB2-mYFP plasmids.
